# Distributed event-triggered robust adaptive formation control of multi-agent rigid bodies on $TSE(3)^N$

**DOI:** 10.1038/s41598-026-47678-1

**Published:** 2026-04-17

**Authors:** Manmohan Sharma, Ramalingam Sakthivel, Kuldeep Yadav

**Affiliations:** 1 School of Electronics Engineering, Vellore Institute of Technology, Kelambakkam-Vandalur Road, Chennai, 600127 Tamil Nadu India; 2 School of Computer Science and Engineering, Vellore Institute of Technology, Kelambakkam-Vandalur Road, Chennai, 600127 Tamil Nadu India

**Keywords:** Formation control, Event-triggered control, Rigid bodies, Bearing vector, Rotation matrix, Engineering, Mathematics and computing, Physics

## Abstract

The study introduces a distributed robust adaptive event-triggered methodology for formation control of multi-agent rigid bodies within the $TSE(3)^N$ framework, utilizing bearing vector measurements. The rigid body’s mass and moment of inertia are treated as unknown but bounded, with an adaptive law designed to estimate these parameters. A time-varying yet bounded disturbance is assumed to influence each rigid body. The robust adaptive control law is adapted to event-triggered scenarios to mitigate communication demands across the network. The communication topology is undirected. A rigorous mathematical proof demonstrates that tracking errors converge exponentially to a residual set. Numerical simulations validate the proposed approach, and a comparative discussion with existing literature is provided.

## Nomenclature

1. $SO(3)=\{R \in \mathbb {R}^{3 \times 3}|RR^T=I_3, det(R)=1\}$ where $I_3$ is the $3 \times 3$ identity matrix.
2. $$SE(3)=\big \{T=\begin{pmatrix}R & p\\ 0 & 1\end{pmatrix} \in \mathbb {R}^{4 \times 4}|R \in SO(3), p \in \mathbb {R}^{3} \big \}$$
3. *TSE*(3) is mathematically defined as the semi-direct product of Lie algebra of SE(3) with itself i.e. $TSE(3)=se(3) \times SE(3)$. Formally, it can be defined as the set of $(R,\Omega ,x,v)$, where $\Omega$ is the angular velocity, *x* is the position vector of the rigid body and *v* is the velocity of the rigid body.

## Introduction

The control of a rigid body is not trivial because the dynamics of the rigid body evolve on a nonlinear manifold *TSE*(3)^1,2^. The attitude control of a rigid body in local coordinates, such as Euler angles, suffers from singularities, while quaternions suffer from ambiguity of representation. To overcome difficulties associated with singularity and ambiguity, the tracking control of a rigid body using rotation matrices has attracted the attention of many researchers^3–9^. All the above-mentioned literature utilizes rotation matrices to represent the attitude of the rigid body. In^3,5,9^, the attitude control is achieved without angular velocity measurement. Attitude control with delayed attitude measurement is presented in^6^ while state constraints are considered in^7^. To add to the difficulty of rigid body control, the parameters of the rigid body (mass and inertia) are not known exactly. Therefore, accurate tracking of a rigid body is a difficult task.

Developing an accurate tracking controller for a rigid body is still an interesting problem because of its several applications in UAVs, spacecraft, manipulators, etc. There are several control algorithms for the operation of a single rigid body. Lately, the research has shifted to control of multiple rigid bodies in several scenarios such as formation control, consensus control, cooperative control, rendezvous, load transportation, etc. The control of multiple rigid bodies is quite challenging simply because we have to control the individual rigid bodies and coordinate between them. There is a growing interest in the research community in developing efficient control algorithms to operate several UAVs in the multiple scenarios mentioned above. This article proposes a solution to distributed formation control of multiple rigid bodies using bearing vector measurements with the event-triggered approach in the presence of parametric uncertainties and bounded disturbances.

A centralized approach to formation control has been proposed in^10^. But due to several advantages such as fault tolerance, scalability, flexibility, etc., a distributed approach has been proposed in^11,12^. Looking at the control mechanism, three different formation control approaches are popular, namely, the behavior-based approach^13^, the virtual structure approach^14^, and the leader-follower approach^15–17^. The comparison between these methods is presented in^18^, but a brief idea about them is presented below. In the behavior-based approach, the desired behavior of each agent is described, and the final control is the weighted sum of each desired behavior. This approach is suitable if the agents have multiple competitive objectives, but this approach is mathematically difficult to analyze, and the stability of formation cannot be guaranteed. In the virtual structure approach, the whole formation is considered as a single structure, which is desirable in certain applications. The final control is derived in multiple steps. However, treating the whole formation as a single unit limits the usefulness of the approach. In leader follower approach, one of the agents is declared leader while others are followers. The control action is derived from following the states of the leader with some offsets. The attractive feature of the leader-follower approach is that the behavior of only the leader needs to be specified. In this paper, we also resort to the leader-follower approach to formation control, where one agent is the leader.

However, the above methods have a singularity in representing the attitude, and hence, the region of convergence is not global or almost global. A consensus algorithm overcoming this singularity problem for rigid bodies has been presented in^19,20^, but the method is limited to attitude control only, and no uncertainty in parameters has been taken into consideration. A formation control algorithm for quadrotor UAVs overcoming singularity problem has been applied in^21,22^, but the problem of communication delay and bandwidth conservation has not been addressed in these papers. A consensus algorithm for Euler-Lagrange systems in the presence of communication delays has been presented in^23^, but the same method cannot be extended to rigid bodies without incurring singularity. The method has been extended to event-triggered scenarios in^24^, but again, the singularity problem is not avoided. A formation control approach for VTOL UAVs in the presence of communication delays has been demonstrated in^25^, but the method utilized quaternions for representing the attitude and hence suffers from the ambiguity of representation.

A formation control method for a swarm of quadrotor UAVs using only position and attitude measurements is studied in^26^, but no method to conserve bandwidth is presented. An adaptive supper twisting sliding mode strategy for formation control of multi-agent systems evolving on the Special Euclidean group is proposed in^27^ with actuator fault and matched uncertainties in the agents? inertia parameters, but the control is not event-triggered. A novel framework for a multi-robot network is proposed to maintain an invariant rigid geometric shape in^28^, but the control is not event-triggered and hence results in inefficient network utilization. Similarly, both distance and bearing constraints are considered in^29^for defining the desired formation, but the method is not event-triggered. A neural network-based event-triggered attack-compensation control problem of T-S fuzzy systems in the presence of actuator attacks is presented in^30^. Many such event-triggered schemes are proposed in literature for power grids^31–33^. Many other articles have also been proposed for complex networks^34^ and also guaranteed cost time-varying formation tracking^35^. In^36^, an event-triggering-based strategy for the global attitude synchronization of a network of rigid bodies is presented, but the position dynamics are not considered. A fully distributed formation control problem for UAVs with external disturbances via an event-triggered approach has been studied in^37^, but the method suffers from a singularity problem. Moreover, the method involves too many parameters to be tuned, which is difficult at times. Similarly, a distributed adaptive dynamic event-triggered formation control protocol is presented in^38^, but again, the method suffers from a singularity problem. Also, the method involves too many parameters to be tuned, which is difficult at times. A robust attitude synchronization of a multi-spacecraft formation system subjected to limited communication, space disturbances, modeling uncertainties, and actuator faults is studied in^39^, but the method is not global since it utilizes quaternions for representing attitude. Similarly, the methods proposed in^40,41^ suffer from singularity problems.

The article proposes a distributed formation control algorithm for multiple rigid bodies using bearing vector measurements in the presence of practical challenges such as bandwidth constraints as well as disturbances. The author has utilized bearing vector measurements since formations obtained using bearing vectors are rigid in any dimensional space. The contributions and novelty of the proposed article are as follows :

1. The formation control algorithm has been developed on $TSE(3)^N$. There are several control algorithms for single and double integrator dynamics^42–53^ considering various state constraints. In^43^, event-triggered consensus for single integrator dynamics has also been considered. However, these are not directly applicable to rigid body dynamics since the rigid body dynamic is nonlinear. The methods utilized for formation control of the rigid body, as mentioned in the above paragraphs, have a singularity. The presence of singularity results in a limited region of operation, which means aggressive maneuvers cannot be performed, which may be necessary in certain formation scenarios. The author presented an algorithm for consensus of multiple rigid bodies on $TSO(3)^N$ in^54^, but that method is not directly applicable to the formation control scenario. Therefore, the author proposes a singularity-free method of formation control of multiple rigid bodies on $TSE(3)^N$ in this article. This is done by representing the attitude of the rigid body by the rotation matrix. This avoids the singularity problem with Euler angles or ambiguity with quaternions.
2. The presence of disturbances cannot be ruled out in mechanical systems operating in real-time scenarios. Moreover, it is very difficult to know the real parameters of the systems. Therefore, the second novelty is that the formation control method has been developed in the presence of parametric uncertainties and external disturbances. The bound on the tracking errors has also been found mathematically. The literature mentioned in point 1 does handle external disturbances, but the methodology is valid only for single and double-integrator dynamics. Therefore, a robust adaptive law has been proposed to handle the disturbances as well as parametric uncertainties directly on $TSE(3)^N$. The robust adaptive law is developed on *TSE*(3) which again avoids singularity issues with Euler angles or ambiguity with quaternions.
3. The third novelty is that an event-triggered approach to formation control has been proposed for multiple rigid bodies in the presence of parametric uncertainties as well as time-varying disturbances. Some of the notable works on event-triggered control of multi-agent systems are^39,55–61^, but these works have either singularity in representation or are not valid for rigid body dynamics. Therefore, an event-triggered approach on $TSE(3)^N$ has been proposed in this article. This approach will not only reduce the communication burden on the network but will also result in power saving and, hence, extended operation time. The event-triggered approach is also developed on *TSE*(3) which avoids singularity issue and ambiguity issue with local representations. A comparison result with^37^ is also presented to show its advantages.
4. Compared to recent works^37,38^, the triggering instants in the proposed method are fewer, and there are fewer parameters to be tuned. This makes the proposed approach simple to implement in practical systems. Moreover, the computational complexity of the proposed approach is much less as demonstrated in Section "Discussion and comparative study". More details can be found in Section "Discussion and comparative study". Compared to another recent work^39^, our method is simple to implement and guarantees almost global convergence.

As per the author’s best knowledge, there is no article in the literature proposing to solve the formation control problem of multiple rigid bodies on $TSE(3)^N$ with an event-triggered approach considering parametric uncertainties as well as disturbances. The proposed article is an attempt to fill this gap and overcome theses shortcomings of the present literature by solving this complex problem on $TSE(3)^N$ with event-triggered approach resulting in fewer trigger instants as demonstrated in comparison section 6. The huge application of such a control algorithm motivated the author to propose this article.

The article is organized into seven sections. The first section “Nomenclature” summarizes the various notations used in the article. The second section “Introduction” presents the literature survey relevant to the method proposed in this article, while the third section “Preliminaries” presents the preliminaries of the concepts utilized for the solution of the problem. The fourth section “Controller design” first proposes a geometric controller for the consensus of rotational dynamics with bounded error, and then a controller is proposed for the formation and control of translational dynamics with bounded errors. The section also proposes the event-triggered controller design for saving transmission resources as well as the stability proof of the complete dynamics. Section “Numerical simulations” presents the numerical simulation result on four multi-agent rigid bodies, with agent-1 being the root node. A comparative study and discussion are presented in Section "Discussion and comparative study". The article is summarized in Section “Conclusion”.

## Preliminaries

### Dynamic model

**Fig. 1:**
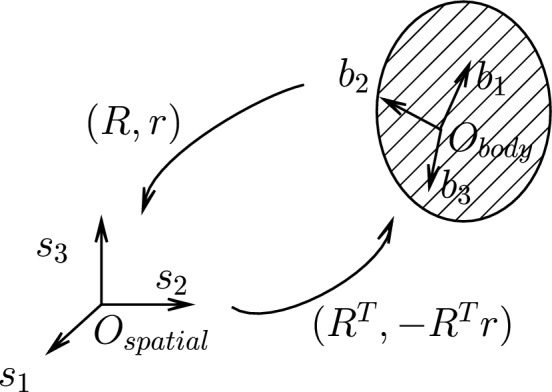
Rigid body.

The rotational dynamics of $i_{th}$ rigid body is given by^62^:

1 $$\begin{aligned}&\dot{R}_i=R_i{\Omega _i}^\dagger , \nonumber \\&J_i\dot{\Omega _i}=-\Omega _i \times J_i\Omega _i+\tau _i+d_{\Omega _i}, \end{aligned}$$

where $R_i$ is the rotation matrix of agent *i* from body-fixed frame $\{O_{body},b_1,b_2,b_3\}$ to inertial frame $\{O_{spatial},s_1,s_2,s_3\}$ as shown in Figure 1. $\Omega _i$ is the angular velocity of agent *i*. $J_i$ is the inertia matrix. $\tau _i$ are the control moments. $d_{\Omega _i}$ is the time-varying disturbance affecting the rotational dynamics of agent *i*. ${(\cdot )}^\dagger$ is a map called hat map from $\mathbb {R}^3$ to $\mathfrak {so}$(3) i.e. if $\Omega =[b_1~~b_2~~b_3]^T$, then

$$\begin{aligned} {\Omega }^\dagger = \left[ \begin{array}{ccc} 0 & -b_3 & b_2 \\ b_3& 0& -b_1\\ -b_2& b_1& 0 \end{array} \right] . \end{aligned}$$

The inverse of the hat map is the vee map $(\cdot )^\vee : \mathfrak {so}(3)$ to $\mathbb {R}^3$. Some of the properties of hat map utilized in this paper is mentioned below :

$$\begin{aligned}&{x}^\dagger y=x\times y=-y \times x. \\&\text {tr}[A{x}^\dagger ]=\frac{1}{2}\text {tr}[{x}^\dagger (A-A^T)]=-x^T(A-A^T)^\vee . \\&{x}^\dagger A+A^T {x}^\dagger ={(\{\text {tr}(A)I_3-A\}x)}^\dagger . \\&R{x}^\dagger R^T={(Rx)}^\dagger . \end{aligned}$$

For some matrix $A \in \mathbb {R}^{3 \times 3}$, $R \in SO(3)$ and $I_3$ is the $3 \times 3$ identity matrix. The translational dynamics of $i_{th}$ rigid body is given by :

2 $$\begin{aligned}&\dot{x}_i=R_iv_i, \nonumber \\&m_i\dot{v}_i=-m_i(\Omega _i \times v_i)+m_igR_i^Te_3+f_i+d_{T_i}, \end{aligned}$$

where, $x_i$ is the position of agent *i* in the inertial-fixed frame of reference while $v_i$ is the velocity of agent *i* in the body-fixed frame of reference. $m_i$ is the mass of the rigid body and $f_i$ is the force in the body-frame. $g=9.81 ~m/s^2$ and $e_3=[0~0~1]^T$. $d_{T_i}$ is the time-varying disturbance affecting the translational dynamics of agent *i*.

### Graph theory

For multiple agents, the interaction between the agents is represented using graph theory. An agent is the vertex of the graph, while an edge is defined as a pair of vertices representing the communication between the agents. If agent *j* can obtain information about agent *i*, the edge is represented as (*i*, *j*). For an undirected graph, the edge (*i*, *j*) means that both the agents *i* and *j* can obtain information from each other. In this article, the communication topology is assumed to be undirected. Let the position vector of agents *i* and *j* are $x_i$ and $x_j$, then the bearing vector $g_{ji}$ is defined as :

$$\begin{aligned} g_{ji}=\frac{x_j-x_i}{||x_j-x_i||}=\frac{z_{ji}}{||z_{ji}||}, \end{aligned}$$

where $z_{ji}=x_j-x_i$. The desired bearing vector from agent *i* to agent *j* is denoted by $g_{ji}^*$ and is defined as:

$$\begin{aligned} g_{ji}^*=\frac{x_j^*-x_i^*}{||x_j^*-x_i^*||}, \end{aligned}$$

where $x_j^*$ and $x_i^*$ are the desired values of $x_j$ and $x_i$ respectively. The orthogonal projection matrix of a non-zero vector *x* is defined as :

$$\begin{aligned} P_x=\mathcal {I}_n-\frac{x}{||x||}\frac{x^T}{||x||}. \end{aligned}$$

### Consensus in rigid body systems

Consider a set of *N* rigid bodies indexed as $i=1,2,\cdots ,N$. The attitude of the rigid bodies are represented by $R_i,i=1,2,\cdots ,N$. The set of rigid bodies are said to achieve consensus if

$$\begin{aligned} ||R_i^TR_j-I_3|| < \epsilon _1 ~~\forall j \in \mathcal {N}_i, \end{aligned}$$

where $\epsilon _1$ is a small positive number and $\mathcal {N}_i$ is the set of neighbors of *i*.

Consider the same set of *N* rigid bodies indexed as $i=1,2,\cdots ,N$. The position and velocity of the rigid bodies are represented by $x_i$ and $v_i,i=1,2,\cdots ,N$ respectively. The set of rigid bodies are said to achieve formation if

$$\begin{aligned} ||x_i-x_j-\delta _{ij}||< \epsilon _2 ~~\forall j \in \mathcal {N}_i \ \text{ and }\ |v_i-v_j|| < \epsilon _3 ~~\forall j \in \mathcal {N}_i, \end{aligned}$$

where $\epsilon _2$, $\epsilon _3$ are small positive numbers and $\delta _{ij}$ represents the desired relative distance between the agents *i* and *j* which may be a constant.

## Controller design

One should note following assumptions before proceeding.

### Assumption 1

^63^ The disturbances affecting the attitude as well as the translational dynamics of each rigid body is bounded i.e. :

$$\begin{aligned}&||d_{\Omega _i}|| \le d_R \ \text{ and }\ ||d_{T_i}|| \le d_T. \end{aligned}$$

### Assumption 2

The communication topology is undirected.

### Assumption 3

^63^ The norm of the inertia matrix as well as the mass of each rigid bodies are unknown but bounded by some known constants.

### Remark 1

It should be noted that all the assumptions are valid in a practical system.

The subsequent section presents a proposal for the stability of the rotational subsystem under bounded error. We demonstrate that the closed-loop subsystem can achieve stability with bounded errors using the adaptive control law for moment of inertia.

### Stability proof for rotational subsystem

#### Proposition 1

The control law (3) along with the adaptation law (4) results in attitude consensus of the rigid bodies with bounded error :

3 $$\begin{aligned}&\tau _i=\Omega _i \times \hat{J}_i \Omega _i-\frac{k_R}{|\mathcal {N}_i|}\sum _{j \in \mathcal {N}_i}e_{R_{ij}}-\frac{k_\Omega }{|\mathcal {N}_i|}\sum _{j \in \mathcal {N}_i}e_{\Omega _{ij}} +\frac{1}{|\mathcal {N}_i|}\hat{J}_i\sum _{j \in \mathcal {N}_i}\dot{\Omega }_j , \end{aligned}$$

4 $$\begin{aligned}&\dot{\hat{J}}_i=-|\mathcal {N}_i|\Omega _i(\sum _{j \in \mathcal {N}_i}(e_{\Omega _{ij}}+e_{R_{ij}}) \times \Omega _i)^T +(\sum _{j \in \mathcal {N}_i} \dot{\Omega }_j)(\sum _{j \in \mathcal {N}_i}(e_{\Omega _{ij}}+e_{R_{ij}}))^T-\sigma \hat{J}_i, \end{aligned}$$

where $\mathcal {N}_i$ is set of neighbors of agent *i* and $|\mathcal {N}_i|$ is the cardinality of this set. $\hat{J}_i$ is the estimate of $J_i$. $k_R,k_\Omega$ and $\sigma$ are the gain matrices.

#### Proof

Let us consider the following Lyapunov function for agent *i*:

5 $$\begin{aligned}&V_{R_i}=\sum _{j \in \mathcal {N}_i} \sum _{k \in \mathcal {N}_i} e_{\Omega _{ij}}^TJ_ie_{\Omega _{ik}}+\frac{1}{2}\sum _{j \in \mathcal {N}_i}\text {tr}(I_3-R_j^TR_i)+c_1\sum _{j \in \mathcal {N}_i} \sum _{k \in \mathcal {N}_i}e_{R_{ij}}^TJ_ie_{\Omega _{ik}}+\frac{1}{2}\text {tr}(\tilde{J}_i^T\tilde{J}_i), \end{aligned}$$

where $e_{\Omega _{ij}}=\Omega _i-R_i^TR_j\Omega _j, e_{R_{ij}}=\frac{1}{2}(R_j^TR_i-R_i^TR_j)^\vee$ and $\tilde{J}_i=J_i-\hat{J}_i$. $()^\vee$ : vee map is inverse of hat map. The last term can also be written as the Frobenius norm of $\tilde{J}_i$ i.e. $||J_i||_F^2$. Then, from Lemma 3, one can write :

6 $$\begin{aligned} \lambda _m(W_1)||z_R||^2 \le V_{R_i} \le \lambda _M(W_2)||z_R||^2, \end{aligned}$$

where $z_R=[||e_{\Omega _{ij}}||~||e_{R_{ij}}||~||\tilde{J}_i||]$, $\lambda _m(\cdot )$ is the minimum eigenvalue of its argument, $\lambda _M(\cdot )$ is the maximum eigenvalue of its argument, $W_1$ and $W_2$ are shown in (7),(8). $W_1$ and $W_2$ can be made positive definite by proper choice of $c_1$, where $c_1$ is a positive constant. Also, $0< c_1 < 1$.

7 $$\begin{aligned} W_1= \left[ \begin{array}{ccc} diag(\lambda _m(J_i))I_{|\mathcal {N}_i| \times |\mathcal {N}_i|} & \frac{c_1}{2}\lambda _m(J_i)I_{|\mathcal {N}_i| \times |\mathcal {N}_i|} & 0 \\ \frac{c_1}{2}\lambda _m(J_i)I_{|\mathcal {N}_i| \times |\mathcal {N}_i|} & diag(\frac{1}{2})I_{|\mathcal {N}_i| \times |\mathcal {N}_i|} & 0 \\ 0 & 0 & diag(\frac{1}{2})I_{|\mathcal {N}_i| \times |\mathcal {N}_i|} \end{array} \right] . \end{aligned}$$

8 $$\begin{aligned} W_2= \left[ \begin{array}{ccc} diag(\lambda _M(J_i))I_{|\mathcal {N}_i| \times |\mathcal {N}_i|} & \frac{c_1}{2}\lambda _M(J_i)I_{|\mathcal {N}_i| \times |\mathcal {N}_i|} & 0 \\ \frac{c_1}{2}\lambda _M(J_i)I_{|\mathcal {N}_i| \times |\mathcal {N}_i|} & diag(\frac{1}{2-\psi })I_{|\mathcal {N}_i| \times |\mathcal {N}_i|} & 0 \\ 0 & 0 & diag(\frac{1}{2})I_{|\mathcal {N}_i| \times |\mathcal {N}_i|} \end{array} \right] . \end{aligned}$$

Taking the time derivative of (5), one gets :

$$\begin{aligned} \dot{V}_{R_i}=&\sum _{j \in \mathcal {N}_i} \sum _{k \in \mathcal {N}_i} e_{\Omega _{ij}}^TJ_i(\dot{\Omega }_i-\dot{\Omega }_k)+\frac{1}{2}\sum _{j \in \mathcal {N}_i}e_{R_{ij}}^Te_{\Omega _{ij}}+c_1\sum _{j \in \mathcal {N}_i} \sum _{k \in \mathcal {N}_i}\dot{e}_{R_{ij}}^TJ_ie_{\Omega _{ik}} \\&+c_1\sum _{j \in \mathcal {N}_i} \sum _{k \in \mathcal {N}_i}\dot{e}_{R_{ij}}^TJ_i(\dot{\Omega }_i-\dot{\Omega }_k)-\text {tr}(\tilde{J}_i^T\dot{\hat{J}}_i), \\ =&\sum _{j \in \mathcal {N}_i} e_{\Omega _{ij}}^TJ_i(|\mathcal {N}_i|\dot{\Omega }_i-\sum _{k \in \mathcal {N}_i}\dot{\Omega }_k)+\frac{1}{2}\sum _{j \in \mathcal {N}_i}e_{R_{ij}}^Te_{\Omega _{ij}} +c_1\sum _{j \in \mathcal {N}_i} \dot{e}_{R_{ij}}^TJ_i\sum _{k \in \mathcal {N}_i}e_{\Omega _{ik}} \\&+c_1\sum _{j \in \mathcal {N}_i}\dot{e}_{R_{ij}}^TJ_i(|\mathcal {N}_i|\dot{\Omega }_i-\sum _{k \in \mathcal {N}_i}\dot{\Omega }_k) -\text {tr}(\tilde{J}_i^T\dot{\hat{J}}_i). \end{aligned}$$

Substituting (1) and (3), one gets :

$$\begin{aligned} \dot{V}_{R_i}=&\sum _{j \in \mathcal {N}_i} e_{\Omega _{ij}}^T(|\mathcal {N}_i|(-\Omega _i \times \tilde{J}_i \Omega _i)-k_R\sum _{k \in \mathcal {N}_i}e_{R_{ik}} -k_\Omega \sum _{k \in \mathcal {N}_i}e_{\Omega _{ik}}+\hat{J}_i\sum _{k \in \mathcal {N}_i}\dot{\Omega }_k -J_i\sum _{k \in \mathcal {N}_i}\dot{\Omega }_k+|\mathcal {N}_i|d_{\Omega _i}) \\&+\sum _{j \in \mathcal {N}_i}e_{R_{ij}}^Te_{\Omega _{ij}}+c_1\sum _{j \in \mathcal {N}_i} \dot{e}_{R_{ij}}^TJ_i\sum _{k \in \mathcal {N}_i}e_{\Omega _{ik}} +c_1\sum _{j \in \mathcal {N}_i} e_{R_{ij}}^T(|\mathcal {N}_i|(-\Omega _i \times \tilde{J}_i \Omega _i)-k_R\sum _{k \in \mathcal {N}_i}e_{R_{ik}}\\&-k_\Omega \sum _{k \in \mathcal {N}_i}e_{\Omega _{ik}}+\hat{J}_i\sum _{k \in \mathcal {N}_i}\dot{\Omega }_k-J_i\sum _{k \in \mathcal {N}_i}\dot{\Omega }_k +|\mathcal {N}_i|d_{\Omega _i})-\text {tr}({\tilde{J}_i}^T\dot{\hat{J}}_i). \end{aligned}$$

From Lemma 1, $||\dot{e}_{R_{ij}}|| \le ||e_{\Omega _{ij}}||$. Therefore, the above equation becomes:

$$\begin{aligned} \dot{V}_{R_i}=&\sum _{j \in \mathcal {N}_i} e_{\Omega _{ij}}^T(-k_R\sum _{k \in \mathcal {N}_i}e_{R_{ik}}-k_\Omega \sum _{k \in \mathcal {N}_i}e_{\Omega _{ik}}+|\mathcal {N}_i|d_{\Omega _i}) +\sum _{j \in \mathcal {N}_i}e_{R_{ij}}^Te_{\Omega _{ij}}+c_1\sum _{j \in \mathcal {N}_i} \dot{e}_{R_{ij}}^TJ_i\sum _{k \in \mathcal {N}_i}e_{\Omega _{ik}} \\&+c_1\sum _{j \in \mathcal {N}_i} e_{R_{ij}}^T(-k_R\sum _{k \in \mathcal {N}_i}e_{R_{ik}} -k_\Omega \sum _{k \in \mathcal {N}_i}e_{\Omega _{ik}}+|\mathcal {N}_i|d_{\Omega _i}) \sum _{j \in \mathcal {N}_i}(e_{\Omega _{ij}}+e_{R_{ij}})^T(|\mathcal {N}_i|(-\Omega _i \times \tilde{J}_i \Omega _i) \\&-J_i\sum _{k \in \mathcal {N}_i}\dot{\Omega }_k) -\text {tr}(\tilde{J}_i^T\dot{\hat{J}}_i) \\ =&-k_\Omega \sum _{j \in \mathcal {N}_i}||e_{\Omega _{ij}}||^2-k_R \sum _{j \in \mathcal {N}_i}||e_{R_{ij}}||^2-c_1\sum _{j \in \mathcal {N}_i}\sum _{k \in \mathcal {N}_i} (k_R e_{\Omega _{ij}}^Te_{R_{ik}}+k_\Omega e_{\Omega _{ij}}^Te_{\Omega _{ik}}+k_R e_{R_{ij}}^Te_{R_{ik}}+k_\Omega e_{R_{ij}}^Te_{\Omega _{ik}} \\&+|\mathcal {N}_i|e_{\Omega _{ij}}^Td_{\Omega _i}+|\mathcal {N}_i|e_{R_{ij}}^Td_{\Omega _i})-|\mathcal {N}_i|\tilde{J}_i\dot{\Omega }^T(\sum _{j \in \mathcal {N}_i}(e_{\Omega _{ij}} +e_{R_{ij}}) \times \Omega _i)-\tilde{J}_i(\sum _{j \in \mathcal {N}_i}(e_{\Omega _{ij}}+e_{R_{ij}})^T)(\sum _{k \in \mathcal {N}_i}\dot{\Omega }_k). \end{aligned}$$

By employing Young’s inequality $a\cdot b \le \frac{||a||^2}{2}+\frac{||b||^2}{2}$, we can write :

9 $$\begin{aligned} \dot{V}_{R_i} \le&-(k_\Omega -c_1\frac{k_\Omega }{2}-c_1\frac{k_R}{2}-\lambda _M(J_i))\sum _{j \in |\mathcal {N}_i|}||e_{\Omega _{ij}}||^2 -(k_R-c_1\frac{k_R}{2}-c_1\frac{k_\Omega }{2}-\lambda _M(J_i))\sum _{j \in |\mathcal {N}_i|}||e_{R_{ij}}||^2 \nonumber \\&-\text {tr}(\tilde{J}_i(|\mathcal {N}_i|)(\sum _{j \in \mathcal {N}_i}(e_{\Omega _{ij}}+e_{R_{ij}})\times \Omega _i)^T +(\sum _{k \in \mathcal {N}_i}\dot{\Omega }_k)(\sum _{j \in \mathcal {N}_i}(e_{\Omega _{ij}}+e_{R_{ij}})\times \Omega _i)^T+\dot{\hat{J}}_i)+||d_R||^2. \end{aligned}$$

From (4), we have

$$\begin{aligned} \dot{V}_{R_i} \le&-(k_\Omega -c_1\frac{k_\Omega }{2}-c_1\frac{k_R}{2}-\lambda _M(J_i))\sum _{j \in |\mathcal {N}_i|}||e_{\Omega _{ij}}||^2 -(k_R-c_1\frac{k_R}{2}-c_1\frac{k_\Omega }{2} \\&-\lambda _M(J_i))\sum _{j \in |\mathcal {N}_i|}||e_{R_{ij}}||^2-\sigma \text {tr}(\tilde{J}_i \hat{J}_i)+||d_R||^2. \end{aligned}$$

The second last term in the right hand side can be written as :

$$\begin{aligned} \tilde{J}_i\hat{J}_i=-\frac{3}{4}\tilde{J}_i^2-(\frac{\tilde{J}_i}{2}-J_i)^2+J_i^2. \end{aligned}$$

Therefore, the last inequality becomes :

10 $$\begin{aligned} \dot{V}_{R_i} \le&-(k_\Omega -c_1\frac{k_\Omega }{2}-c_1\frac{k_R}{2}-\lambda _M(J_i))\sum _{j \in |\mathcal {N}_i|}||e_{\Omega _{ij}}||^2 -(k_R-c_1\frac{k_R}{2}-c_1\frac{k_\Omega }{2}-\lambda _M(J_i))\sum _{j \in |\mathcal {N}_i|}||e_{R_{ij}}||^2 \nonumber \\&-\frac{3\sigma }{4}\text {tr}(\tilde{J}_i^2)-\sigma \text {tr}(\frac{\tilde{J}_i}{2}-J_i)^2+\sigma \text {tr}(J_i^2)+||d_R||^2. \end{aligned}$$

Let $z_R=[||e_{\Omega _{ij}}||~||e_{R_{ij}}||~||\tilde{J}_i||]$, then the above inequality becomes :

11 $$\begin{aligned} \dot{V}_{R_i} \le&-\lambda _m(W)||z_R||^2-\sigma \text {tr}(\frac{\tilde{J}_i}{2}-J_i)^2+\sigma \text {tr}(J_i^2)+||d_R||^2, \nonumber \\ \le&-\lambda _m(W)||z_R||^2+\sigma \text {tr}(J_i^2)+||d_R||^2, \nonumber \\ \le&-\frac{\lambda _m(W)}{\lambda _M(W_2)}V_R+\sigma \text {tr}(J_i^2)+||d_R||^2, \end{aligned}$$

where $W=\text {diag}(k_\Omega -c_1\frac{k_\Omega }{2}-c_1\frac{k_R}{2}-\lambda _M(J_i),k_R-c_1\frac{k_R}{2}-c_1\frac{k_\Omega }{2}-\lambda _M(J_i),\frac{3\sigma }{4})$ which implies that if $V_R \ge V_{R_0}=\frac{\lambda _m(W_2)}{\lambda _M(W)}(\sigma \text {tr}(J_i^2)+||d||^2)$, then $\dot{V}_R \le 0$. In addition we can establish by integrating the above inequality that $||z_R||$ converges exponentially to the residual set:

12 $$\begin{aligned} D_{R_i}=\bigg \{||z_R|| \in \mathbb {R} \bigg | ||z_R||^2 \le \frac{\sigma \text {tr}(J_i^2)+||d||^2}{\lambda _m(W)}\bigg \}. \end{aligned}$$

### Event-triggered control for attitude dynamics

If the control input is event-triggered then we can take $\tau _i$ as

$$\begin{aligned} \tau _i=\tau _t+e_{t_R}, \end{aligned}$$

where $\tau _t=\tau _i$ at the triggering instant and $\tau _t(k+1)=\tau _t(k)$ if *k* is not the triggering instant. $\tau _t$ is selected as in Proposition 1 at the triggering instant and the triggering instant is decided by the violation of the following inequality :

13 $$\begin{aligned} \dot{V}_{R_e}=&-k_\Omega \sum _{j \in \mathcal {N}_i}||e_{\Omega _{ij}}||^2-k_R \sum _{j \in \mathcal {N}_i}||e_{R_{ij}}||^2-c_1\sum _{j \in \mathcal {N}_i}\sum _{k \in \mathcal {N}_i} (k_R e_{\Omega _{ij}}^Te_{R_{ik}}+k_\Omega e_{\Omega _{ij}}^Te_{\Omega _{ik}}\nonumber \\&+k_R e_{R_{ij}}^Te_{R_{ik}}+k_\Omega e_{R_{ij}}^Te_{\Omega _{ik}} +e_{\Omega _{ij}}^T{e_{t_R}}+e_{R_{ij}}^T{e_{t_R}}) \le 0. \end{aligned}$$

In other words, the triggering instant for $i_{th}$ rigid body is defined as :

$$\begin{aligned} t_{k+1}^i=inf\bigg \{t>t_k^i\big |\dot{V}_{R_e}> 0\bigg \}. \end{aligned}$$

If $V_R \ge V_{R_0}$, then it can be easily seen that this triggering mechanism renders the attitude errors bounded. Between the triggering instants the previous received values of states are used in evaluating (13). When this inequality is violated the states are exchanged and control inputs updated to stabilize the attitude dynamics. $\square$

In the next section, the stability of the translational subsystem with bounded error is proposed. It is shown that, in the presence of the adaptive control law for mass, the control action renders the closed-loop subsystem stable with bounded error.

### Stability proof for translational subsystem

#### Proposition 2

The control law (14) along with the adaptation law (15) results in formation tracking of the rigid bodies with bounded error :

14 $$\begin{aligned}&f_i=\hat{m}_i(\Omega _i \times v_i)+\hat{m}_igR_i^Te_3 -\frac{k_v}{|\mathcal {N}_i|}\sum _{j \in \mathcal {N}_i}e_{v_{ij}}-\frac{k_p}{|\mathcal {N}_i|}\sum _{j \in \mathcal {N}_i}\frac{P_{g{ij}}}{||z_{ij}||}g_{ij}^*+\frac{1}{|\mathcal {N}_i|}\hat{m}_i\sum _{j \in \mathcal {N}_i}\dot{v}_j, \end{aligned}$$

15 $$\begin{aligned}&\dot{\hat{m}}_i=-|\mathcal {N}_i|\Omega _i(\sum _{j \in \mathcal {N}_i}(e_{v_{ij}}+e_{p_{ij}}))^T(gR_i^Te_3+\Omega _i \times v_i)-(\sum _{j \in \mathcal {N}_i}(e_{v_{ij}}+e_{p_{ij}}))^T(\sum _{k \in \mathcal {N}_i}\dot{v}_k)-\gamma \hat{m}_i, \end{aligned}$$

where $\hat{m}_i$ is the estimate of $m_i$. $k_v,k_p$ and $\gamma$ are the gain matrices.

#### Proof

Let the Lyapunov function for the translational subsytem be :

16 $$\begin{aligned}&V_{T_i}=\frac{1}{2}\sum _{j \in \mathcal {N}_i} \sum _{k \in \mathcal {N}_i} e_{v_{ij}}^Tm_ie_{v_{ik}}+\frac{1}{2}\sum _{j \in \mathcal {N}_i}||g_{ij}-g_{ij}^*||^2+c_2\sum _{j \in \mathcal {N}_i} \sum _{k \in \mathcal {N}_i}\bigg (\frac{P_{g{ij}}}{||z_{ij}||}g_{ij}^*\bigg )^Tm_ie_{v_{ik}}+\frac{1}{2}\tilde{m}_i^2, \end{aligned}$$

where, $e_{v_{ij}}=v_i-v_j, e_{p_{ij}}=\frac{P_{g{ij}}}{||z_{ij}||}g_{ij}^*$ and $\tilde{m}_i=m_i-\hat{m}_i$. From Lemma 4, one can write :

17 $$\begin{aligned} \lambda _m(W_3)||z_T||^2 \le V_{T_i} \le \lambda _M(W_4)||z_T||^2, \end{aligned}$$

where $z_T=[||e_v||~||e_p||~|\tilde{m}_i|]$, $W_3$ and $W_4$ are shown in (18),(19). $W_3$ and $W_4$ can be made positive definite by proper choice of $c_2$, where $c_2$ is a constant. Also, $0< c_2 < 1$. Taking the time derivative of (16), one gets:

18 $$\begin{aligned} W_3= \left[ \begin{array}{ccc} m_iI_{|\mathcal {N}_i| \times |\mathcal {N}_i|} & \frac{c_2}{2}m_i I_{|\mathcal {N}_i| \times |\mathcal {N}_i|} & 0 \\ \frac{c_2}{2}m_i I_{|\mathcal {N}_i| \times |\mathcal {N}_i|} & \frac{1}{2}I_{|\mathcal {N}_i| \times |\mathcal {N}_i|} & 0 \\ 0 & 0 & \frac{1}{2}I_{|\mathcal {N}_i| \times |\mathcal {N}_i|} \end{array} \right] . \end{aligned}$$

19 $$\begin{aligned} W_4= \left[ \begin{array}{ccc} m_iI_{|\mathcal {N}_i| \times |\mathcal {N}_i|} & \frac{c_2}{2}m_i I_{|\mathcal {N}_i| \times |\mathcal {N}_i|} & 0 \\ \frac{c_2}{2}m_i I_{|\mathcal {N}_i| \times |\mathcal {N}_i|} & \frac{1}{1-\beta }I_{|\mathcal {N}_i| \times |\mathcal {N}_i|} & 0 \\ 0 & 0 & \frac{1}{2}I_{|\mathcal {N}_i| \times |\mathcal {N}_i|} \end{array} \right] . \end{aligned}$$

$$\begin{aligned} \dot{V}_{T_i}=&\sum _{j \in \mathcal {N}_i} \sum _{k \in \mathcal {N}_i} e_{v_{ij}}^Tm_i(\dot{v}_i-\dot{v}_k)+\sum _{j \in \mathcal {N}_i}e_{v_{ij}}^Te_{p_{ij}}+c_2\sum _{j \in \mathcal {N}_i} \sum _{k \in \mathcal {N}_i}\dot{e}_{p_{ij}}^Tm_ie_{v_{ik}}+c_2\sum _{j \in \mathcal {N}_i} \sum _{k \in \mathcal {N}_i}e_{p_{ij}}^Tm_i(\dot{v}_i-\dot{v}_k)-\tilde{m}_i^T\dot{\hat{m}}_i \\ =&\sum _{j \in \mathcal {N}_i} e_{\Omega _{ij}}^Tm_i(|\mathcal {N}_i|(-m_i(\Omega _i \times v_i)-m_igR_i^Te_3+f_i +d_{T_i})-\sum _{k \in \mathcal {N}_i}\dot{\Omega }_k)+\sum _{j \in \mathcal {N}_i}e_{v_{ij}}^Te_{p_{ij}}+c_2\sum _{j \in \mathcal {N}_i} \dot{e}_{p_{ij}}^Tm_i\sum _{k \in \mathcal {N}_i}e_{v_{ik}} \\&+c_2\sum _{j \in \mathcal {N}_i}e_{p_{ij}}^Tm_i(|\mathcal {N}_i(-m_i(\Omega _i \times v_i)-m_igR_i^Te_3+f_i +d_{T_i})-\sum _{k \in \mathcal {N}_i}\dot{v}_k)-\tilde{m}_i^T\dot{\hat{m}}_i. \end{aligned}$$

Substituting the control law (14) and the adaptive law (15) in the above equation, we get:

$$\begin{aligned} \dot{V}_{T_i}=&\sum _{j \in \mathcal {N}_i} e_{v_{ij}}^T(|\mathcal {N}_i|(-\tilde{m}_i(\Omega _i \times v_i)-\tilde{m}_igR_i^Te_3)- k_v\sum _{k \in \mathcal {N}_i}e_{v_{ik}}-k_p\sum _{k \in \mathcal {N}_i}e_{p_{ik}}+\hat{m}_i\sum _{k \in \mathcal {N}_i}\dot{v}_k-m_i\sum _{k \in \mathcal {N}_i}\dot{v}_k \\&+|\mathcal {N}_i|d_{T_i})+\sum _{j \in \mathcal {N}_i}e_{v_{ij}}^Te_{p_{ij}}+c_2\sum _{j \in \mathcal {N}_i} \dot{e}_{p_{ij}}^Tm_i\sum _{k \in \mathcal {N}_i}e_{v_{ik}}+c_2\sum _{j \in \mathcal {N}_i} e_{p_{ij}}^T(|\mathcal {N}_i|(-\tilde{m}_i(\Omega _i \times v_i)-\tilde{m}_igR_i^Te_3) \\&-k_v\sum _{k \in \mathcal {N}_i}e_{v_{ik}}-k_p\sum _{k \in \mathcal {N}_i}e_{p_{ik}}+\hat{m}_i\sum _{k \in \mathcal {N}_i}\dot{v}_k-m_i\sum _{k \in \mathcal {N}_i}\dot{v}_k +|\mathcal {N}_i|d_{T_i})-\tilde{m}_i^T\dot{\hat{m}}_i. \end{aligned}$$

From Lemma 2, $||\dot{e}_{p_{ij}}|| \le ||e_{v_{ij}}||$. Therefore, the above equation becomes :

$$\begin{aligned} \dot{V}_{T_i} \le&-k_v \sum _{j \in \mathcal {N}_i}||e_{v_{ij}}||^2-k_p \sum _{j \in \mathcal {N}_i}||e_{p_{ij}}||^2+c_2\sum _{j \in \mathcal {N}_i} \sum _{k \in \mathcal {N}_i}(k_v e_{v_{ij}}^Te_{v_{ik}}+k_p e_{v_{ij}}^Te_{p_{ik}}+k_v e_{p_{ij}}^Te_{v_{ik}}+k_p e_{p_{ij}}^Te_{p_{ik}} \\&+|\mathcal {N}_i|e_{v_{ij}}^Td_{T_i}+|\mathcal {N}_i|e_{p_{ij}}^Td_{T_i})+\sum _{j \in \mathcal {N}_i}(e_{v_{ij}}+e_{p_{ij}})^T (|\mathcal {N}_i|(\tilde{m}_igR_i^Te_3-\tilde{m}_i(\Omega _i \times v_i)-\tilde{m}_i\sum _{k \in \mathcal {N}_i}\dot{\Omega }_k)-\tilde{m}_i\dot{\hat{m}}_i. \end{aligned}$$

By employing Young’s inequality, we can write :

$$\begin{aligned} \dot{V}_{T_i} \le&-(k_v-c_2\frac{k_v}{2}-c_2\frac{k_p}{2}-m_i)\sum _{j \in |\mathcal {N}_i|}||e_{v_{ij}}||^2 -(k_p-c_2\frac{k_v}{2}-c_2\frac{k_p}{2}-m_i)\sum _{j \in |\mathcal {N}_i|}||e_{p_{ij}}||^2 \\&-\tilde{m}_i(|\mathcal {N}_i|)(\sum _{j \in \mathcal {N}_i}(e_{v_{ij}}+e_{p_{ij}})^T(gR_i^Te_3+\Omega _i \times v_i) +(\sum _{k \in \mathcal {N}_i}\dot{v}_k)+\dot{\hat{m}}_i)+||d_{T_i}||^2. \end{aligned}$$

From (15), we have :

$$\begin{aligned} \dot{V}_{T_i} \le&-(k_v-c_2\frac{k_v}{2}-c_2\frac{k_p}{2}-m_i)\sum _{j \in |\mathcal {N}_i|}||e_{v_{ij}}||^2 -(k_R-c_2\frac{k_p}{2}-c_2\frac{k_v}{2}-m_i)\sum _{j \in |\mathcal {N}_i|}||e_{p_{ij}}||^2 +\gamma \tilde{m}_i \hat{m}_i+||d_{T}||^2. \end{aligned}$$

The second last term in the right hand side can be written as :

$$\begin{aligned} \tilde{m}_i\hat{m}_i=-\frac{3}{4}\tilde{m}_i^2-(\frac{\tilde{m}_i}{2}-m_i)^2+m_i^2. \end{aligned}$$

Therefore, the last inequality becomes :

$$\begin{aligned} \dot{V}_{T_i} \le&-(k_v-c_2\frac{k_v}{2}-c_2\frac{k_p}{2}-m_i)\sum _{j \in |\mathcal {N}_i|}||e_{v_{ij}}||^2 -(k_p-c_2\frac{k_v}{2}-c_2\frac{k_p}{2}-m_i)\sum _{j \in |\mathcal {N}_i|}||e_{p_{ij}}||^2 \\&-\frac{3\gamma }{4}\tilde{m}_i^2-\gamma (\frac{\tilde{J}_i}{2}-m_i)^2+\gamma m_i^2+||d_{T}||^2. \end{aligned}$$

Let $z_T=[||e_v||~||e_p||~|\tilde{m}_i|]$, then the above inequality becomes :

20 $$\begin{aligned} \dot{V}_{T_i} \le&-\lambda _m(W_5)||z_T||^2-\gamma (\frac{\tilde{m}_i}{2}-m_i)^2+\gamma m_i^2+||d_{T}||^2, \nonumber \\&\le -\lambda _m(W_5)||z_T||^2+\gamma m_i^2+||d_{T}||^2, \nonumber \\&\le -\frac{\lambda _m(W_5)}{\lambda _M(W_4)}V_T+\gamma m_i^2+||d_{T}||^2, \end{aligned}$$

where $W_5=\text {diag}(k_v-c_2\frac{k_v}{2}-c_2\frac{k_p}{2}-m_i,k_p-c_2\frac{k_v}{2}-c_2\frac{k_p}{2}-m_i,\frac{3\gamma }{4})$ which implies that if $V_T \ge V_{T_0}=\frac{\lambda _m(W_5)}{\lambda _M(W_4)}(\gamma m_i^2+||d_{T_i}||^2)$, then $\dot{V}_T \le 0$. In addition we can establish by integrating the above inequality that $||z_R||$ converges exponentially to the residual set :

21 $$\begin{aligned} D_{T_i}=\bigg \{||z_T|| \in \mathbb {R} \bigg | ||z_T||^2 \le \frac{\gamma m_i^2+||d_{T_i}||^2}{\lambda _m(W_5)}\bigg \}. \end{aligned}$$

### Event-triggered control for translational dynamics

If the control input is event-triggered then we can take $\tau _i$ as

22 $$\begin{aligned} f_i=f_t+e_{t_T}, \end{aligned}$$

where $f_t=f_i$ at the triggering instant and $f_t(k+1)=f_t(k)$ if *k* is not the triggering instant. $f_t$ is selected as in Proposition 2 at the triggering instant and the triggering instant is decided by the violation of following inequality :

23 $$\begin{aligned} \dot{V}_{T_e}=&-k_v \sum _{j \in \mathcal {N}_i}||e_{v_{ij}}||^2-k_p \sum _{j \in \mathcal {N}_i}||e_{p_{ij}}||^2-c_2\sum _{j \in \mathcal {N}_i}\sum _{k \in \mathcal {N}_i} (k_v e_{v_{ij}}^Te_{p_{ik}}+k_p e_{v_{ij}}^Te_{p_{ik}}+k_v e_{p_{ij}}^Te_{v_{ik}}\nonumber \\&+k_p e_{p_{ij}}^Te_{p_{ik}} +e_{v_{ij}}^T{e_{t_T}}+e_{p_{ij}}^T{e_{t_T}} \le 0. \end{aligned}$$

In other words, the triggering instant for $i_{th}$ rigid body is defined as :

24 $$\begin{aligned} t_{k+1}^i=inf\bigg \{t>t_k^i\big |\dot{V}_{T_e}> 0\bigg \}. \end{aligned}$$

If $V_T \ge V_{T_0}$, then it can be easily seen that this triggering mechanism renders the translational errors bounded. Between the triggering instants the previous received values of states are used in evaluating (23). When this inequality is violated the states are exchanged and control inputs updated to stabilize the formation. $\square$

### Stability of complete dynamics

Since the rotational and translational dynamics of individual rigid bodies are coupled, it is necessary to prove the stability of the both dynamics together. This can be proved by taking the Lyapunov function as :

25 $$\begin{aligned} V_i=V_{T_i}+V_{R_i}. \end{aligned}$$

Taking the time derivative of (25) and simplifying using (11) and (20), one can conclude that the complete dynamics exponentially converges to the set

$$\begin{aligned} D_{c_i}=D_{R_i} \cup D_{T_i}. \end{aligned}$$

The system dynamics of all the multi-agent rigid bodies will converge to the set

$$\begin{aligned} D_{c}=\bigcap _{i=1}^N D_{c_i}. \end{aligned}$$

where *N* is the number of agents. This can be deduced by taking the Lyapunov function as :

$$\begin{aligned} V=\sum _{i=1}^N V_i. \end{aligned}$$

**Fig. 2:**
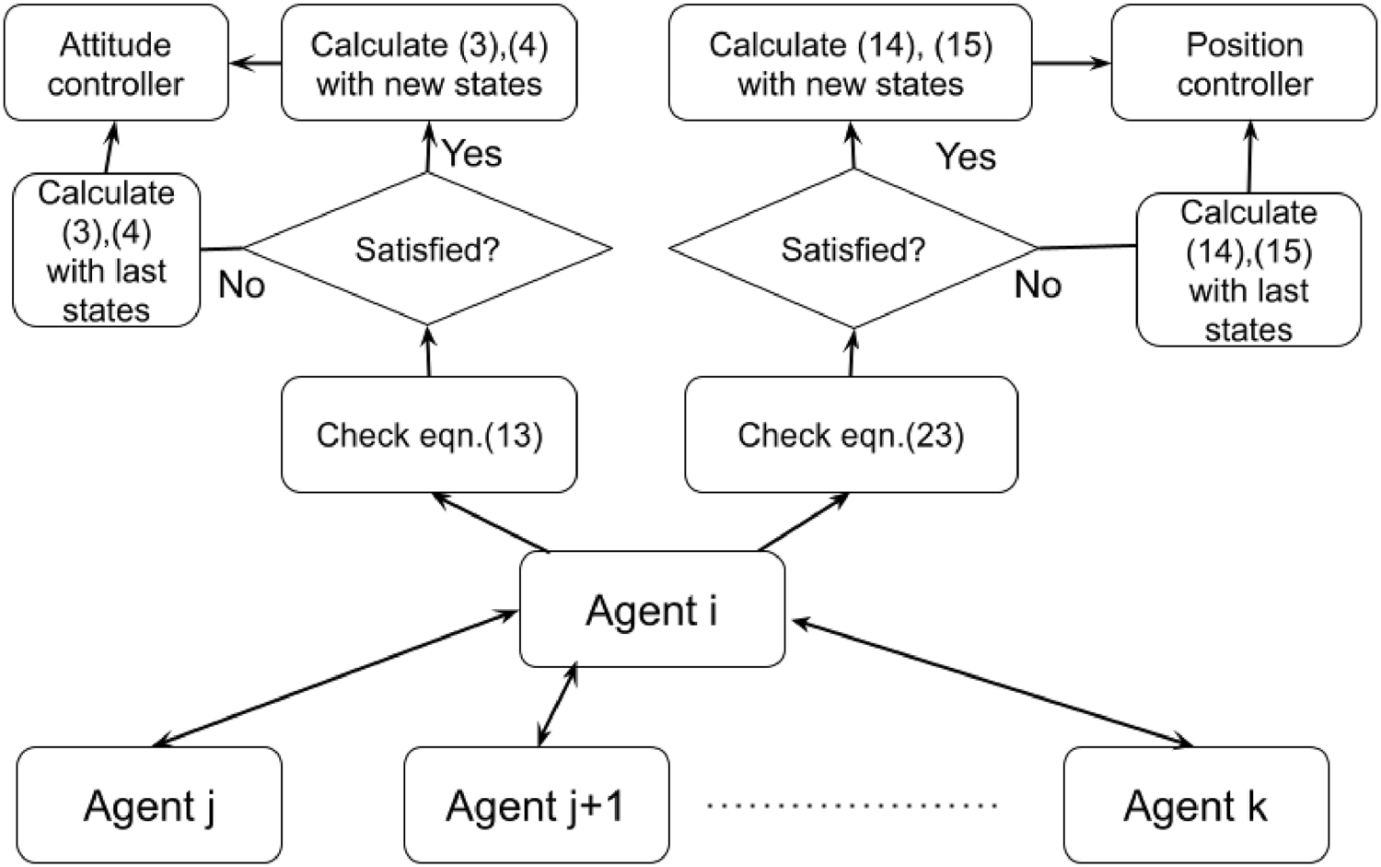
Flowchart of the proposed method.

A flowchart of steps to be followed when implementing the controller is shown in Fig. 2.

### Exclusion of Zeno behaviour

In this section it will be shown that a lower bound on inter-execution time exist under the proposed event-triggered scheme. Let us consider the following derivative

$$\begin{aligned} \frac{d}{dt}|e_{t_R}|=\text {sign}e_{t_R}|\dot{e}_{t_R}| \le |\dot{\tau _i}|, \end{aligned}$$

where $$e_{t_R}=\tau -\tau _t$$ and $$\tau _t$$ is constant in the interval $$[t_k,t_{k+1})$$. It can be seen that $$|\dot{\tau _i}| \le \epsilon$$ for some $$\epsilon> 0$$ since it is a function of bounded signals. Since $$e(t_k)=0$$ and $$lim_{t \rightarrow t_{k+1}}e(t)=\rho$$ for some $$\rho> 0$$, the lower bound on inter-execution time is found as $$\frac{\rho }{\epsilon }$$. This completes the proof for rotational dynamics. Similar proof can be obtained for translational dynamics by considering the derivative of $$|e_{t_T}|$$.

#### Remark 2

Compared to recent works^37,38^, the triggering instants in the proposed method are fewer, and there are fewer parameters to be tuned. This makes the proposed approach simple to implement in practical systems. Moreover, the computational complexity of the proposed approach is much less.

#### Remark 3

The method proposed in^39^ suffers from singularity issues since it utilizes quaternions for representing attitude. Compared to this, our method is singularity-free, is simple to implement, and guarantees almost global convergence.

## Numerical simulations

**Fig. 3:**
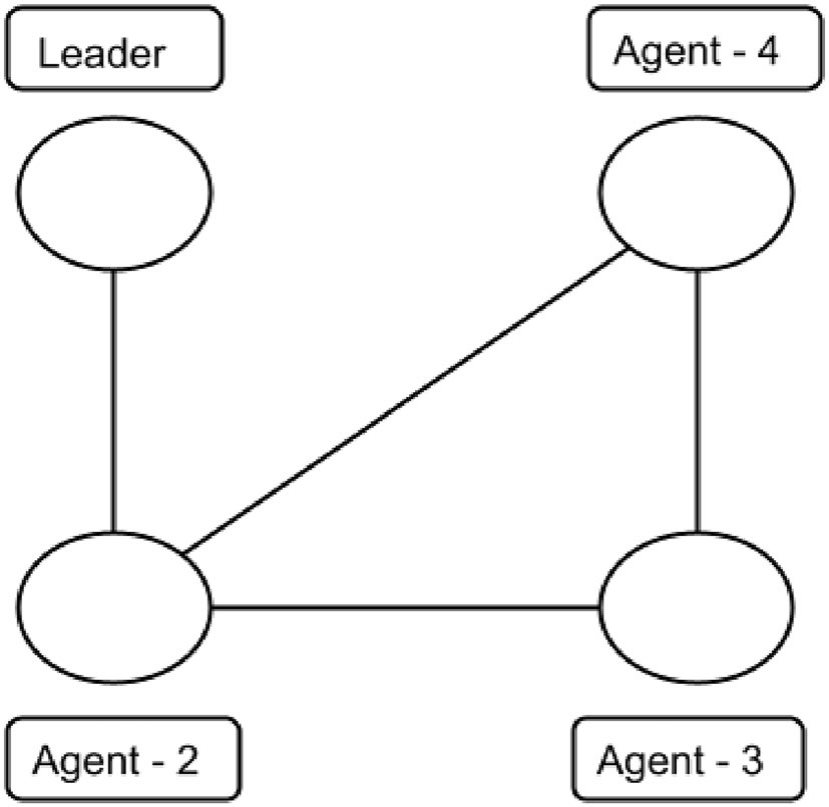
Topology.

A numerical simulation was performed in MATLAB to demonstrate the performance of the adaptive controller proposed in this paper. Four homogeneous rigid bodies were considered in this simulation, and the parameters are listed in the Table. 1. The communication topology is shown in Figure 3, where a line from agent$-i$ to agent$-j$ means that the agent$-i$ is able to access the states of agent$-j$. All the initial velocities were taken to be zero, while the initial positions are mentioned below :

$$\begin{aligned} x_1(0)=[2~0~0]^T,~~x_2(0)=[2~-1~1]^T,~~x_3(0)=[3~-1~1]^T,~~ x_4(0)=[3~0~1]^T. \end{aligned}$$

Therefore, the desired bearing vector can be calculated as :

$$\begin{aligned} g_{ji}^*=\frac{x_j(0)-x_i(0)}{||x_j(0)-x_i(0)||}. \end{aligned}$$

The agent 1 is the leader of the group whose desired trajectory was specified to be

$$\begin{aligned}&x_{1_d}=\bigg [2\cos \bigg (\frac{2 \pi t}{20}\bigg )~~2\sin \bigg (\frac{2 \pi t}{20}\bigg )~~1\bigg ]^T. \end{aligned}$$

**Fig. 4:**
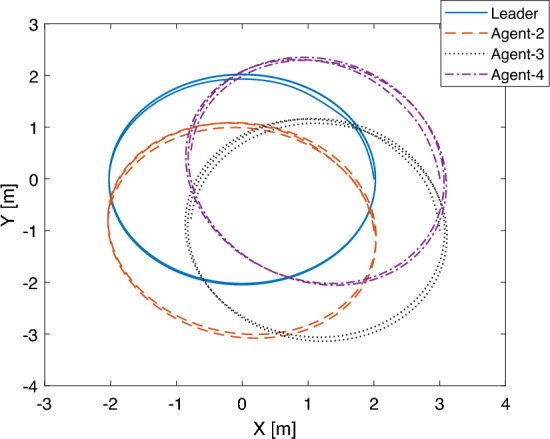
Formation.

**Fig. 5:**
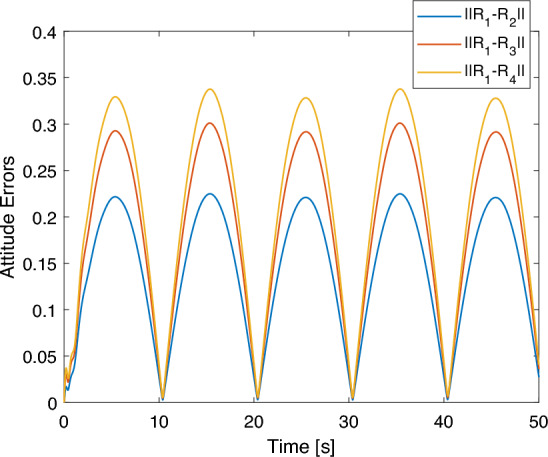
Attitude Synchronization error.

**Fig. 6:**
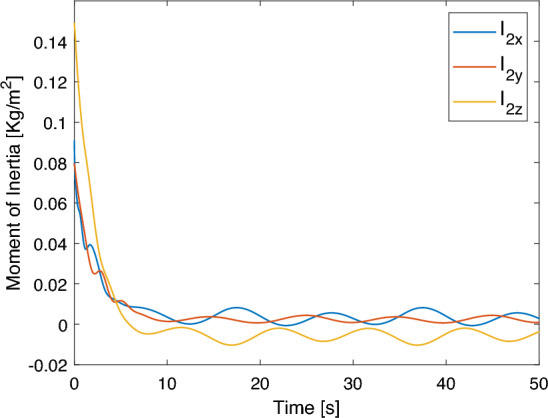
Estimates of moment of inertia.

**Fig. 7:**
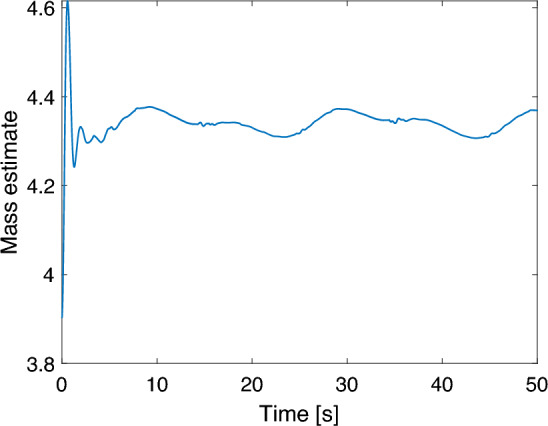
Estimate of mass.

The various plots are shown in Figures 4,5,6,7. The simulation was performed for 50 sec with a step size of 0.01 sec for individual rigid bodies. The communication between the agents was event-triggered. When the conditions of (13) or (23) were violated, the states were exchanged between those agents. The simulation was performed in the presence of time-varying disturbances in both the attitude as well as translational dynamics of each agent. The expression of the disturbance is given below :

$$\begin{aligned} d_{R_i}=[0.2;0.3;0.1]*sin(0.1\pi t), \ d_{T_i}=[0.5;0.4;0.3]*sin(0.1\pi t). \end{aligned}$$

**Fig. 8:**
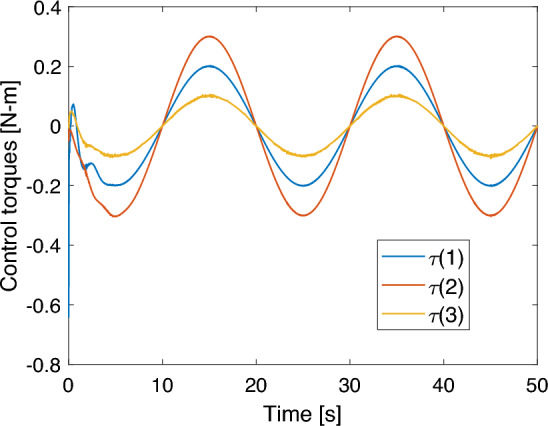
Control torque.

**Fig. 9:**
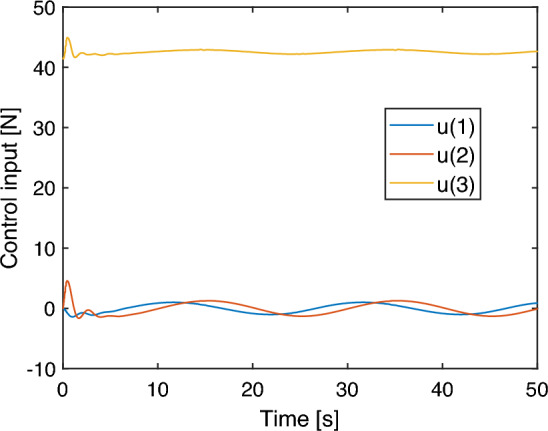
Control thrust.

From the figures, one can observe that the formation is achieved with bounded errors in the presence of time-varying disturbances. The attitude errors are also bounded, as shown in Figure5. The estimate of the moment of inertia as well as mass is also bounded. It is observed from Figure 7 that the estimate of mass varies around the actual mass value. The control inputs are plotted in Figure 8 and Figure 9 from which we can observe that the control inputs are bounded and continuous as desired. The simulation result was given for one agent, but similar results were obtained for all the agents and, hence, are omitted. The number of triggering instants between each agent is mentioned in Table 2. One can observe from the tables that the number of triggers is considerably less than the time-triggered scenario. The number of triggering instances is less than 50$\%$ of the time-triggered scenario. This results in considerable savings of both transmission bandwidth and power. A video presenting the simulation result is available at https://youtu.be/-sJ93JcxT-4.

**Table 1:** Parameters used in simulation.

Parameter	Values	Units	Parameter	Values	Units
*g*	9.81	m/s$^2$	$I_{x}$	0.082	kg m$^{2}$
$m_i$	4.34	kg	$I_{y}$	0.0845	kg m$^{2}$
*h*	0.01	s	$I_{z}$	0.1377	kg m$^{2}$
$k_R$	5	-	$k_v$	5	-
$k_p$	5	-	$K_\Omega$	2	-

**Table 2:** Number of triggers.

Node	No of triggers
$2-1,2-3$	1947
$3-2$	1135
$4-3,4-2$	702

**Fig. 10:**
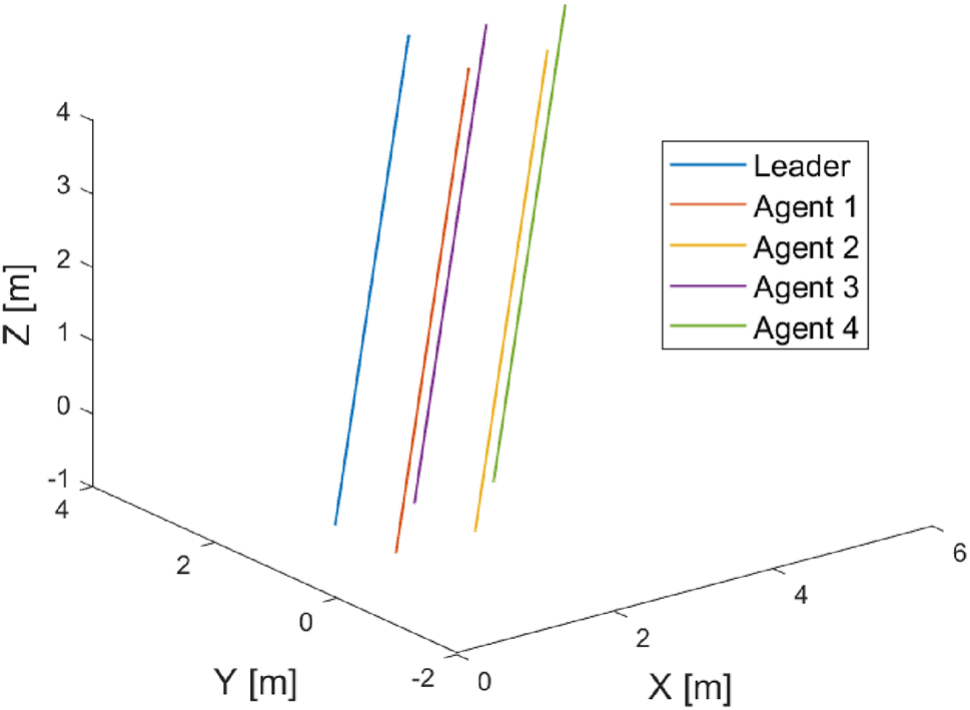
Trajectory of the agents.

## Discussion and comparative study

**Fig. 11:**
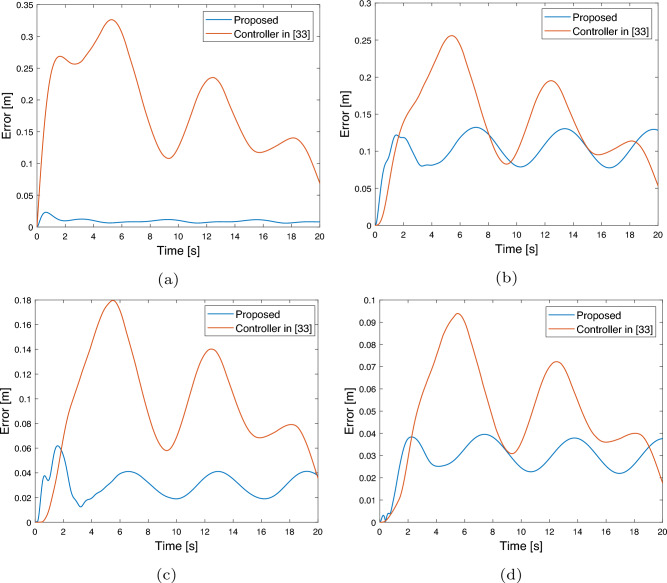
Formation error in position between agents (**a**) $0-1$ (**b**) $1-2$ (**c**) $2-3$ (**d**) $3-4$.

**Fig. 12:**
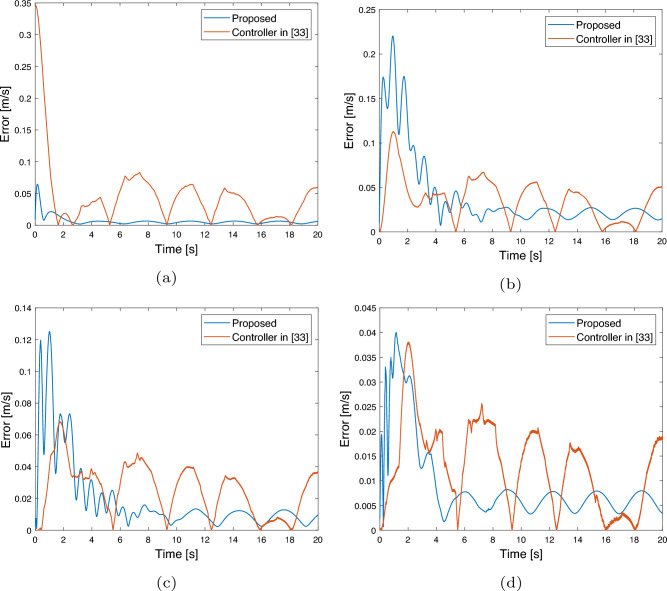
Formation error in velocity between agents (**a**) $0-1$ (**b**) $1-2$ (**c**) $2-3$ (**d**) $3-4$.

A comparison of the proposed approach with^37^ has been presented in this section. The comparison has been done with the same parameters as in^37^. The trajectory of the leader and the agents using the proposed approach is shown in Figure 10. The comparison result is shown in Figures11 and 12. The Figure 11 show the error in relative position between the different agents while the Figures 12 show the error in relative velocity. The quantitative comparison is shown in Tables 3 and 4. Table 3 shows the comparison between the number of triggers between the agents, from which one can infer that the proposed approach results in fewer triggers. Similarly, from Table 4, one can observe that the errors in relative position and velocity between the agents are less in the proposed approach as compared to^37^. This results in tighter formation in the proposed approach. Hence, one can conclude that the proposed approach results in a better formation control strategy with fewer triggers between the agents. Moreover, in our proposed approach, the computational complexity is also less compared to^37,38^. Clearly, in our approach, the number of multiplications required is 16 times the number of neighbors, and the number of additions required is only 14. In^37^, more than 25 multiplications at each node are required, and more than 14 additions need to be performed. In our approach, integration is not required, while in^37^, three integrations per step are needed, which is very exhaustive. Similarly, in^38^, more than 30 multiplications, 20 additions, and three integrations per step are required, which is, again, very exhaustive. Therefore, one can conclude that the proposed approach is better in terms of computational complexity. One can also observe that there are many parameters to be tuned in^37,38^ for whose optimal values may be difficult to find, which is not the case in the proposed approach since there are only a few parameters to be found.

**Table 3:** Comparison of no of triggers.

Node	No of triggers in the proposed method	Controller in^33^
$0-1$	1001	970
$1-2$	939	1421
$2-3$	894	2452
$3-4$	892	1246

**Table 4:** Comparison between RMS errors.

Agents	Error in position in the proposed method [m]	Error in position in^46^ [m]	Error in velocity in the proposed method [m]	Error in velocity in^46^ [m]
$0-1$	0.0099	0.2043	0.0099	0.0705
$1-2$	0.0099	0.2043	0.0099	0.0705
$2-3$	0.0099	0.2043	0.0099	0.0705
$3-4$	0.0099	0.2043	0.0099	0.0705

## Conclusion

An adaptive control law was developed for formation control of multi-agent rigid bodies on $TSE(3)^N$ in the presence of parametric uncertainty as well as external disturbances. Further, an event-triggered algorithm was also developed for formation control, which resulted in considerable savings in transmission bandwidth and power. Mathematical proof was also given to show that formation is achieved in the presence of parametric uncertainty as well as time-varying disturbances. A comparison result with the literature is also given, which shows that the proposed approach is better. The limitation of the present work is that it is valid for undirected topology only. Therefore, future work will focus on designing a formation control algorithm for multi-agent rigid bodies in the presence of communication noise and time delays using directed topology.

## Data Availability

The datasets used and/or analysed during the current study available from the corresponding author on reasonable request.

## Appendix

### Lemma 1

26 $$\begin{aligned} ||\dot{e}_{R_{ij}}|| \le ||e_{\Omega _{ij}}||, \end{aligned}$$

where, $$e_{\Omega _{ij}}=\Omega _i-R_i^TR_j\Omega _j, e_{R_{ij}}=\frac{1}{2}(R_j^TR_i-R_i^TR_j)^\vee$$.

### Proof

$$\begin{aligned} \dot{e}_{R_{ij}} =&\frac{1}{2}(-{\Omega }^\dagger _jR_j^TR_i+R_j^TR_i {\Omega }^\dagger _i+{\Omega }_i^\dagger R_i^TR_j-R_i^TR_j{\Omega }^\dagger _j)^\vee \\ =&\frac{1}{2}(R_j^TR_i{(\Omega _i-R_i^TR_j\Omega _j)}^\dagger +{(\Omega _i-R_i^TR_j\Omega _j)}^\dagger R_i^TR_j)^\vee \\ =&\frac{1}{2}(R_j^TR_i {e}^\dagger _{\Omega _{ij}}+{e}^\dagger _{\Omega _{ij}}R_i^TR_j)^\vee \\ =&\frac{1}{2}(tr(R_i^TR_j)-R_i^TR_j)e_{\Omega _{ij}}\\ =&C(R_j^TR_i)e_{\Omega _{ij}}. \end{aligned}$$

It can be proved that $||C(R_j^TR_i)|| \le 1$ and this proves the lemma. $\square$

### Lemma 2

27 $$\begin{aligned} ||\dot{e}_{p_{ij}}|| \le ||e_{v_{ij}}||, \end{aligned}$$

where, $$e_{v_{ij}}=v_i-v_j, e_{p_{ij}}=\frac{P_{g{ij}}}{||z_{ij}||}g_{ij}^*$$.

### Proof

28 $$\begin{aligned} e_{p_{ij}}=\frac{P_{g{ij}}}{||z_{ij}||}g_{ij}^*=\frac{g_{ij}^*-g_{ij}g_{ij}^Tg_{ij}^*}{||z_{ij}||}. \end{aligned}$$

The derivative of this expression is :

29 $$\begin{aligned} \dot{e}_{p_{ij}}=\frac{\begin{matrix}||z_{ij}||^3(z_{ij}-z_{ij}^Tg_{ij}^*I_3-z_{ij}{g_{ij}^*}^T) -3(||z_{ij}||^2-z_{ij}z_{ij}^T)g_{ij}^*||z_{ij}||^2\frac{z_{ij}}{||z_{ij}||}\end{matrix}}{||z_{ij}||^4}R_ie_{v_{ij}}=D(z_{ij})e_{v_{ij}}. \end{aligned}$$

It can be proved that $||D(z_{ij})|| \le 1$ and this proves the lemma. $\square$

### Lemma 3

Let

30 $$\begin{aligned} \Psi _{ij}=\frac{1}{2}\text {tr}(I_3-R_j^TR_i). \end{aligned}$$

Then one should note following properties of $\Psi _{ij}$.

1. $\Psi _{ij}$ is locally positive definite about $R_i=R_j$.
2. $\Psi _{ij}$ is locally quadratic for $\Psi _{ij}< \psi < 2$ i.e.

31 $$\begin{aligned} \frac{1}{2}||e_{R_{ij}}||^2 \le \Psi _{ij} \le \frac{1}{2-\psi }||e_{R_{ij}}||^2 \end{aligned}$$

for $\psi$ a positive constant.

### Proof

Please see^62^ $$\square$$

### Lemma 4

Let

32 $$\begin{aligned} \Gamma _{ij}=||g_{ij}-g_{ij}^*||^2 \end{aligned}$$

then $\Gamma _{ij}$ is locally quadratic for $g_{ij}^Tg_{ij}^*> -\beta$ and $||z_{ij}|| < \kappa$ i.e.

33 $$\begin{aligned} ||e_{p_{ij}}||^2 \kappa ^2 \le \Gamma _{ij} \le \frac{2}{1-\beta }||e_{p_{ij}}||^2 \kappa ^2. \end{aligned}$$

For $0<\beta <1$ and $\kappa$, positive constants.

### Proof

First observe that,

34 $$\begin{aligned} \Gamma _{ij}=||g_{ij}-g_{ij}^*||^2=2(1-g_{ij}^Tg_{ij}^*). \end{aligned}$$

Also,

35 $$\begin{aligned} ||e_{p_{ij}}||^2=e_{p_{ij}}^Te_{p_{ij}}=\frac{{g_{ij}^*}^TP_{g_{ij}}g_{ij}^*}{||z_{ij}||^2}, \end{aligned}$$

where the second equality comes from the property of projection matrices that is $P^2=P$. Substituting

36 $$\begin{aligned} P_{g_{ij}}=I_n-g_{ij}g_{ij}^T. \end{aligned}$$

one gets :

37 $$\begin{aligned} ||e_{p_{ij}}||^2=1-(g_{ij}^Tg_{ij}^*)^2=(1+g_{ij}^Tg_{ij}^*)\frac{\Gamma _{ij}}{2{||z_{ij}||^2}}. \end{aligned}$$

Therefore,

38 $$\begin{aligned} \Gamma _{ij}=\frac{2||e_{p_{ij}}||^2 ||z_{ij}||^2}{(1+g_{ij}^Tg_{ij}^*)}. \end{aligned}$$

For $g_{ij}^Tg_{ij}^*(t_0)> -\beta$ and $||z_{ij}(t_0)|| \le \kappa$ one has :

39 $$\begin{aligned} ||e_{p_{ij}}||^2 \kappa ^2 \le \Gamma _{ij} \le \frac{2}{1-\beta }||e_{p_{ij}}||^2 \kappa ^2. \end{aligned}$$

Here $t_0$ is the initial time. From Proposition 2, we know that $||g_{ij}||$ is bounded, therefore, $||z_{ij}||$ will also be bounded for all time and hence the above inequality is valid for all time. $\square$
